# Seroprevalence of Anti-SARS-CoV-2 IgG Antibodies in Healthcare Personnel in El Salvador Prior to Vaccination Campaigns

**DOI:** 10.3390/idr16030040

**Published:** 2024-06-07

**Authors:** José Elías Aguilar Ramírez, Adrianna Maliga, Allison Stewart, Allison Lino, José Eduardo Oliva, Xochitl Sandoval, Emily Zielinski-Gutierrez, Rafael Chacon-Fuentes, Parminder S. Suchdev, Susana Zelaya, Mario Sánchez, Delmy Lisseth Recinos, Beatriz López, Ella Hawes, Julie Liu, Shannon E. Ronca, Sarah M. Gunter, Kristy O. Murray, Rhina Domínguez

**Affiliations:** 1 El Salvador National Institute of Health, San Salvador 1101, El Salvador; jelias.aguilar@salud.gob.sv (J.E.A.R.); joseduardoliva67@gmail.com (J.E.O.); susana.zelaya@salud.gob.sv (S.Z.); marioalejandro.sg@gmail.com (M.S.); delmy.recinos@salud.gob.sv (D.L.R.); rhina.dominguez@salud.gob.sv (R.D.); 2 Division of Pediatric Tropical Medicine, Department of Pediatrics, Baylor College of Medicine and Texas Children’s Hospital, Houston, TX 77030, USA; adrianna.maliga@bcm.edu (A.M.); allison.lino@uth.tmc.edu (A.L.); ella.e.hawes@uth.tmc.edu (E.H.); julie.liu@uth.tmc.edu (J.L.); shannon.ronca@bcm.edu (S.E.R.); sarah.gunter@bcm.edu (S.M.G.); 3 William T. Shearer Center for Human Immunobiology, Texas Children’s Hospital, Houston, TX 77030, USA; 4 Centers for Disease Control and Prevention, Central America Office, Guatemala City 01015, Guatemala; allisonlstew@gmail.com (A.S.); ebz0@cdc.gov (E.Z.-G.); qin8@cdc.gov (R.C.-F.); dvo8@cdc.gov (P.S.S.); fdx8@cdc.gov (B.L.); 5 Department of Pediatrics and Children’s Healthcare of Atlanta, Emory University, Atlanta, GA 30033, USA

**Keywords:** COVID-19, SARS-CoV-2, seroprevalence, spike protein, El Salvador

## Abstract

COVID-19, caused by the SARS-CoV-2 virus, is a highly pathogenic emerging infectious disease. Healthcare personnel (HCP) are presumably at higher risk of acquiring emerging infections because of occupational exposure. The prevalence of COVID-19 in HCP is unknown, particularly in low- to middle-income countries like El Salvador. The goal of this study was to determine the seroprevalence of anti-SARS-CoV-2 antibodies among HCP in El Salvador just prior to vaccine rollout in March 2021. We evaluated 2176 participants from a nationally representative sample of national healthcare institutions. We found 40.4% (*n* = 880) of the study participants were seropositive for anti-spike protein antibodies. Significant factors associated with infection included younger age; living within the central, more populated zone of the country; living in a larger household (≥7 members); household members with COVID-19 or compatible symptoms; and those who worked in auxiliary services (i.e., housekeeping and food services). These findings provide insight into opportunities to mitigate SARS-CoV-2 risk and other emerging respiratory pathogens in HCP in El Salvador.

## 1. Introduction

Coronavirus Disease 2019 (COVID-19) is a highly infectious disease caused by the SARS-CoV-2 virus. The clinical syndrome caused by SARS-CoV-2 ranges from very mild symptoms to severe pneumonia, acute respiratory distress syndrome (ARDS), and death [1]. Prior to vaccine rollout, case fatality rates ranged from 7% globally to 17% among a sub-group of hospital-admitted cases, and 40.5% among intensive care cases [2]. By February 2021, El Salvador had reported 162,755 cases of SARS-CoV-2, though the true burden of disease was likely higher [3]. Several studies have found asymptomatic infections to be common [4,5,6]; therefore, the exact number of people who have been infected with SARS-CoV-2 is unknown. In low- to middle-income countries (LMICs), case counts can underrepresent the true burden of infections due to diagnostic limitations and challenges [4].

Healthcare personnel (HCP) are the frontline workforce providing clinical care for confirmed or suspected cases of COVID-19. Consequently, they are presumably at higher risk of acquiring the infection than the general population and, if they become infected, represent a risk of infection for patients, co-workers, household members, and their community [5,6,7]. Early in the pandemic in the United States, up to 19% of COVID-19 cases were found to be diagnosed in healthcare workers [8]; however, the true prevalence of COVID-19 in this risk group is unknown, particularly in LMIC settings [9].

The quantification and characterization of SARS-CoV-2 infection in HCP is unknown in most countries affected by the pandemic, including El Salvador. Serological surveys are useful for inferring the dynamics of a population’s risk of acquiring infection, contributing to improved prevention and mitigation measures through the rapid identification of cases and contacts [10,11]. In this study, we had the opportunity to conduct a country-wide serosurvey of HCP of the National Integrated Health System (El Sistema Nacional Integrado de Salud, SNIS) in El Salvador just prior to the launch of vaccination campaigns in February and March of 2021. The objectives of this study were to determine the prevalence of IgG against SARS-CoV-2 spike protein, describe the clinical and epidemiological characteristics of HCP study participants, and determine factors associated with infection. The goal of this study was to identify opportunities to mitigate risk in this unique and vulnerable population. 

## 2. Materials and Methods

### 2.1. Study Design and Study Population

This study was a nationally representative cross-sectional study among randomly selected HCP employed within the SNIS of El Salvador. The study was conducted between January and February 2021. 

The SNIS is made up of eight institutions: Salvadoran Social Security Institute (ISSS); Solidarity Fund for Health (FOSALUD); Ministry of National Defense referred to the Military Health Command (COSAM); Salvadoran Institute of Magisterial Welfare (ISBM); Salvadoran Institute of Integral Rehabilitation (ISRI); Higher Public Health Council (CSSP); Directorate of Medicines (DNM) and the National Directorate of Higher Education of the Ministry of Education, Science and Technology (MINED) [12]. The SNIS has a total of 1494 healthcare facilities (HCFs) with 47,395 employees.

### 2.2. Sample Size Calculation

We used SNIS categories of job function to define four strata of HCP: medical personnel, nursing staff, technical staff, and administrative staff. We calculated the sample size using the following parameters: estimated prevalence of COVID-19 seropositivity of 50% with a 95% confidence interval, sample error of 5%, estimated population size of 47,395 employees, and a design effect (DEFF) of 1.5. This gave us a sample size of 571 employees in each stratum and combining the four strata resulted in a total sample size of 2284 participants.

### 2.3. Stages of Sample Size Determination

**Figure 1:**
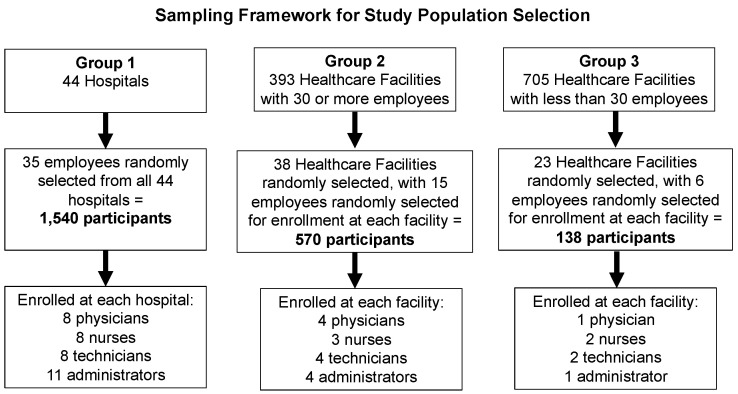
Geospatial map of anti-SARS-CoV-2 IgG antibody seropositivity by district.

In the first stage of sample determination, we classified the SNIS HCFs into three groups by number of employees: group one (44 hospitals), group two (393 HCFs with 30 or more employees), and group three (705 HCFs with less than 30 employees) (Figure 1). We included a random sample of 35 employees from each of the 44 hospitals in group one, which are considered frontline workforce, for a total of 1540 participants. We randomly selected 38 HCFs from group two, then randomly selected 15 employees from each, for a total of 570 participants. Lastly, we randomly sampled 23 HCFs from group three, then randomly selected six employees from each for a total of 138 participants. To maintain the representativeness of the sample, the technical team readjusted the total sample size to 2248 HCP from SNIS HCFs.

Secondly, we determined the number of HCFs and the number of participants in each group by calculating the distribution of each HCF type based on the representativeness of each stratum: physicians (21.4%), nurses (23.6%), technicians (24.1%), and administrative personnel (30.9%). Administrative personnel also included those in auxiliary services, i.e., housekeeping, food, and transport services (Supplemental Table S3). The final distribution by HCF type comprised hospitals (eight physicians, eight nurses, eight technicians, and eleven administrative personnel), HCFs with 30 or more employees (four physicians, three nurses, four technicians, and four administrative personnel), and HCFs with less than 30 employees (one doctor, two nurses, two technicians, and one administrative staff). During the interview process, we further defined their patient contact risk by categorizing the participants into primary job functions as follows: direct patient care, administrative, auxiliary services (i.e., housekeeping, food, and transport services), and contact with patient specimens.

### 2.4. Participant Selection and Enrollment

To sample within each HCF, we conducted systematic participant selection with a random start-up. We included both male and female participants if they were actively working in an SNIS institution, providing direct care to patients, and worked between March and December of 2020 in the selected HCFs. During the interview process, we further defined their patient contact risk by categorizing the participants into primary job functions as follows: direct patient care, administrative, auxiliary services (i.e., housekeeping, food, and transport services), and contact with patient specimens. We excluded those eligible if they did not agree to participate in the study; were in house isolation, quarantine, and or isolation during the pandemic; were hospitalized at the time of data collection; did not present the scheduled day and time for their survey; or were students or trainees. If an individual was eligible for participation but refused or was ineligible, study staff moved on to the next person on the list. After screening, study staff provided electronic consent and completed an electronic questionnaire using the Kobo Toolbox. Questionnaire included sociodemographic information (sex, age, household size, residence), employment information (workplace, level of care of the HCF, HCF location, profession), clinical data (self-reported signs and symptoms in the three months prior to the study, symptom onset date) and medical history (blood type, pregnancy, and history of chronic diseases as detailed in Table 1), and laboratory testing (history of SARS-CoV-2 RT-PCR test and antibody test).

### 2.5. Specimen Collection and Antibody Testing for SARS-CoV-2

We collected serum samples from 2177 HCP and shipped them to Baylor College of Medicine (BCM) for spike protein IgG ELISA testing for antibodies against SARS-CoV-2 using InBios SCoV-2 Detect™ IgG ELISA kits according to the manufacturer’s protocol. The kit has a sensitivity of 91.89% and a specificity of 98.95% [13]. Positive, negative, and cutoff controls were provided to ensure the integrity of the test and to determine the specific threshold of the assay, with 1.1 and higher considered positive and 0.9–1.1 considered equivocal. All specimens with equivocal findings were further analyzed by plaque reduction neutralization tests (PRNTs) to confirm the presence of neutralizing antibodies and confirm seropositivity. 

### 2.6. Data Collection and Statistical Analysis

Interview and testing results were entered, quality checked, and processed using SPSS v. 25, (Chicago, IL, USA), and final data analysis was performed using SAS Studio v. 3.8 (Cary, NC, USA). We characterized the population according to demographic variables, work, clinics, and laboratories. We then calculated the positivity rate of the spike protein SARS-CoV-2 antibodies in HCP along with 95% Wald confidence intervals and distributed by demographic variables, workplace variables, and clinical variables. We then examined the data to determine associations between the presence of spike protein SARS-CoV-2 IgG antibodies and factors associated with infection using bivariable and multivariable analyses and adjusted for the two-stage cluster sampling design. We used statistical significance (*p* < 0.05) in the bivariable analysis and background knowledge of the study team to guide variable selection. We used a correlation matrix to confirm no multicollinearity of variables in the multivariable analysis and analyzed log-likelihood values to compare regression models and determine the goodness of fit for the multivariable model. Variables in the multivariable model included: age group, geographic zone, number of household family members, primary job function, SARS-CoV-2 symptoms, and symptoms in household family members. 

## 3. Results

### 3.1. Characteristics of the Study Population

We enrolled 2177 HCP between January and February of 2021. One was found to be indeterminate on testing for SARS-CoV-2 antibodies; therefore, we excluded this individual from further analysis, giving us a total population of 2176. Stratification by HCF type and job function can be found in Supplemental Table S1. Table 1 presents the sociodemographic characteristics of the enrolled sample. The median age of participants was 43 years with a range from 18 to 84 years of age. A total of 62.9% of participants were female. About one-third of the participants (34.0%) resided in the department of San Salvador. Approximately half of the participants (48.7%) reported having one to three household family members, while 44.4% had four to six household family members. The majority of participants (62.0%) reported their primary job function as providing attention to patients, one quarter (24.5%) were in administrative roles, and the remaining participants worked in auxiliary services, which included housekeeping, food, and transport services. With regards to comorbid conditions, almost one quarter (23.5%) reported having arterial hypertension, 8.5% had diabetes, and 9.3% had obesity.

### 3.2. Estimated Seropositivity by Demographic, Workplace, and Clinical Characteristics

**Figure 2:**
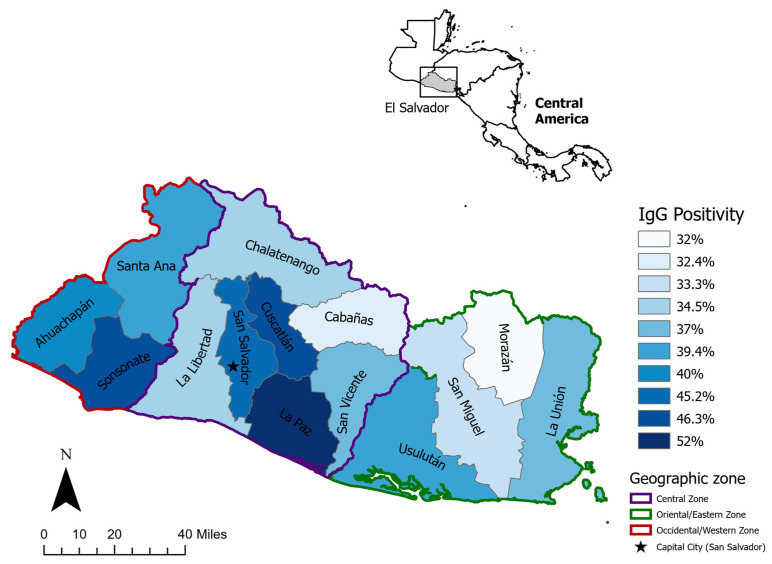
Geospatial map of anti-SARS-CoV-2 IgG antibody seropositivity by district.

Table 2 presents the estimated seropositivity of anti-SARS-CoV-2 IgG antibodies in the study sample and the bivariable analysis for seropositivity, adjusted for the two-stage cluster sampling design and distributed by demographic, workplace, and clinical characteristics. The total estimated seropositivity for the sample was 40.4% (95% CI 38.3–42.6%) with no statistically significant difference in seropositivity between males (40.0% (95% CI 36.8–43.4%)) and females (40.7% (95% CI 38.0–43.3%)). Compared to the estimated seropositivity of participants aged 31–50 (41.8% (95% CI 39.0–44.6%)), higher seropositivity was found among participants younger than 31 (44.4% (95% CI 39.0–49.8%)) and a statistically significant lower odds of seropositivity was found among those aged 51–70 years compared to those aged 31–50 years (OR: 0.77 (95% CI 0.64–0.93)). Seropositivity was higher among participants residing in the Central zone (42.2% (95% CI 39.4–45.0%)) compared to those in the Western (41.1% (95% CI 37.0–45.3%)) and Eastern (35.2% (95% CI 30.6–39.7%)) zones (Figure 2). Compared to participants with no household family members, there were significantly higher odds of seropositivity among participants with seven or more household family members (OR: 2.13 (95% CI 1.1–4.3)) and non-significantly higher odds among participants with one to three household family members (OR: 1.15 (95% CI 0.6–2.1)) and four to six household family members (OR: 1.45 (95% CI 0.8–2.7)).

No statistically significant difference in seropositivity was found by primary job function. Compared to participants who provided direct patient care as their primary job function, participants who worked in auxiliary services had significantly higher odds of seropositivity (OR: 1.48 (95% CI 1.10–1.99)) and non-significantly lower odds were found for those who worked in administrative roles (OR: 0.81 (95% CI 0.65–1.02)) and specimen handling (OR: 0.72 (95% CI 0.49–1.04)). No statistically significant difference in seropositivity was found when comparing participants who held only one job (40.5% (95% CI 38.3–42.7)) to those who held two or more jobs (39.5% (95% CI 29.5–49.4)).

Seropositivity among those who had previously received a positive RT-PCR result (66.1% (95% CI 62.0–70.2%)) was significantly higher than among those who had not received a positive result (28.0% (95% CI 25.6–30.3%); OR: 5.03 (95% CI 4.05–6.24)). Participants who reported having family members with SARS-CoV-2 symptoms before developing their own illness had significantly higher odds of seropositivity compared to participants who did not (OR: 1.61 (95% CI 1.33–1.96)), and participants who had household family members diagnosed with SARS-CoV-2 had significantly higher odds of seropositivity compared to participants who did not have household family members with SARS-CoV-2 (OR: 2.72 (95% CI 2.21–3.33)).

Table 3 shows SARS-CoV-2 IgG seropositivity by varying symptoms that were experienced in the prior three months. The highest seropositivity was found among those who had experienced loss of taste (*n* = 138; 85.5% seropositive (95% CI 79.3–91.8%)) and was associated with nearly 10 times increased odds of testing positive (OR: 9.89 (95% CI 5.96–16.40)). Seropositivity among those who reported having no symptoms was 36.2% (95% CI 33.6–38.9%), while significantly higher odds of seropositivity were found among those who had two or more symptoms (OR: 1.62 (95% CI 1.33–1.96)) compared to those who reported no symptoms. No statistically significant difference in seropositivity was found when comparing participants who experienced one symptom (41.6% (95% CI 35.5–47.6%)) with those who experienced no symptoms (36.2% (95% CI 33.6–38.9%)).

### 3.3. Multivariable Analysis of Factors Associated with Infection

As shown in Table 4, after adjusting for all other covariates, there were significantly lower odds of seropositivity among participants aged 51–70 years compared to those aged 31–50 (OR: 0.81 (95% CI 0.67–0.99)) and among those residing in the Eastern zone compared to the Central zone (OR: 0.71 (95% CI 0.56–0.90)). Significantly higher odds of seropositivity were found among those who had seven or more household family members compared to those who had zero household family members (OR: 2.56 (95% CI 1.20–5.46)) and among those who worked in auxiliary services support compared to those providing attention to patients (OR: 1.56 (95% CI 1.12–2.18)), adjusting for all other covariates. Finally, participants who reported experiencing two or more symptoms had significantly higher seropositivity compared to participants who reported zero symptoms (OR: 1.49 (95% CI 1.21–1.83)). Participants who had household family members with symptoms had significantly higher seropositivity compared to participants who had no household family members with symptoms (OR: 1.51 (95% CI 1.22–1.86)), adjusting for all other covariates.

## 4. Discussion

With the imminent launch of COVID-19 vaccination campaigns, we took the opportunity to understand the impact of natural infection in the first year of the pandemic among HCP, a critical frontline and high-risk group [6]. We found that four out of ten HCP had anti-spike protein SARS-CoV-2 antibodies, with no significant differences by sex. Statistically significant factors associated with infection included younger age; living within the central, more populated zone of the country; living in a larger household (≥7 members); household members with COVID-19 or compatible symptoms; and those who worked in auxiliary services. These findings support the importance of community transmission in disease spread. Although there was no nationwide study conducted in El Salvador to compare the results, the prevalence found was higher compared to systematic reviews of the prevalence found in the general populations of other countries [14,15]. For instance, a worldwide systematic review of seroprevalence studies among the general population from January 2020 to March 2021 found a global SARS-CoV-2 seroprevalence of 9.5% [14]. Another systematic review and meta-analysis among HCP found a weighted average of 8% seropositivity [16]. In this review, having a high number of household contacts was a factor associated with seropositivity, as was found in our study. The review also found higher seroprevalence associated with males, inconsistent with our findings, and with ethnicity, which was not included in our investigation due to the homogenous nature of the population.

We found significantly decreased seropositivity in participants aged 51–70 years versus those aged 31–50 years. These findings may be attributed to national mandates implemented in May 2020 that aimed to reduce the risk of exposure among vulnerable populations, requiring people with chronic conditions to stay at home, reducing their risk of SARS-CoV-2 infection, along with public health campaigns targeting those at risk for severe disease, particularly older populations [17]. As a result, older individuals were likely more vigilant in protecting themselves against infection. Additionally, more than 70% of the sample belonged to the age group of 50 years or younger, which could be related to a sampling bias due to younger HCP working at the HCFs at the time of enrollment. This could have contributed to the increased positive cases among the younger age groups and the limitations in our results.

The protective effect of residing in the Eastern zone compared to the Central zone may be attributed to the difference in population density between the two zones. Decreased population density in the Eastern zone could result in fewer contacts and a lower risk of transmission of SARS-CoV-2, both in and outside of work environments [18] Living with more than seven people and having family members with symptoms were factors associated with increased seropositivity, which could be explained in a similar manner due to increased risk of transmission within the participants’ homes. 

Finally, significantly higher seropositivity was found in those working in auxiliary services, such as those that clean the facilities, materials, and equipment, or prepare and provide food to patients. One explanation for this association may be that those who are part of auxiliary services rotate units and interact with different areas of the HCFs, possibly increasing their risk of exposure to COVID-19-positive patients. Another explanation could be a lack of personal protective equipment (PPE) or a lack of adequate use of PPE, increasing the risk of infection. Previous studies have cited the shortage of PPE as a risk factor for death from SARS-CoV-2 among HCP [19]. One review of HCP in 11 African countries found a lack of education and training in infection control measures to be associated with seropositivity in this group [20]. Additionally, there has been varying evidence around the differential risk of SARS-CoV-2 infection by type of healthcare function—it is not exclusive to those who provide patient care. Transmission of SARS-CoV-2 has been reported in clinical and administrative staff in HCFs [21,22,23], long-term care homes [24,25], and outpatient services [26], affecting a wide range of technicians, nurses, and physicians [27,28,29]. One study reported higher rates of SARS-CoV-2 transmission in nurses, while physicians were at higher risk of death. This study also found being frontline HCP (direct contact with confirmed or suspected COVID-19 patients in emergency, hospitalization, or intensive care departments, as well as those involved in the transfer of these patients) as a factor associated with the highest risk of death [30]. However, other studies found a similar risk of infection, regardless of the function or location of HCP [31]. Our finding of increased seropositivity among personnel in auxiliary roles points to a unique risk of SARS-CoV-2 infection for these personnel. 

While we hypothesize that the increased seropositivity among personnel in auxiliary services could have been linked to a lack of PPE or training around infection control measures, our study did not obtain information on PPE use among the participants, limiting the conclusiveness of this hypothesis. Other limitations of the study included lack of data on COVID-19 severity, the cross-sectional design that limits analysis of acute infection and changes in the levels of antibodies over time, limiting the ability to know exact rates of infection prior to vaccine rollout in El Salvador. Additionally, small samples from each HCF limited the representativeness of these institutions and the comparison of participants by HCF. Our sampling strategy was focused on ensuring the representativeness of HCP across the national healthcare system in El Salvador, hence we did not include non-HCP in the community. Future studies could be designed to focus on comparing the risk of infection in HCP versus the general population. We were unable to conduct RT-PCR tests to test for acute infections and were not able to compare current versus past infections in participants. Finally, while the sensitivity of the InBios SCoV-2 Detect™ IgG ELISA kits is quite high at 91.89% [13], there is still the possibility of missing a small sample of true positive cases. Similarly, if the participant was within just a few days of the acute phase of infection, IgG antibodies might not have had the time to develop. Should future resources permit additional testing, then we could test all samples with a second antibody assay and confirm all positives by PRNT. 

Our study is the first national seroprevalence survey among HCP in El Salvador. We were able to achieve a nationally representative sample, including participants from a variety of HCFs and diverse job positions within these facilities. This contributes to the generalizability of our results to HCP throughout El Salvador. Additionally, the timing of data collection before vaccination campaigns allows us to conclusively state that seropositivity among these participants was a result of natural infection and not antibody development from the SARS-CoV-2 vaccines.

## 5. Conclusions

Our nationally representative seroprevalence study contributes to an improved understanding of the seroprevalence of a highly pathogenic emerging respiratory pathogen, SARS-CoV-2, among HCP in El Salvador prior to vaccine rollout. The results of our study can help inform preventive measures taken to limit exposure among HCP, such as improvements to the provision and training on the proper use of PPE, especially among those who do not have direct patient contact. The associations we found with higher numbers of household members and with personnel in auxiliary roles suggest increased transmission of SARS-CoV-2 in the community outside the HCFs. Future studies can expand on our findings by exploring modes of SARS-CoV-2 transmission among HCP outside of direct patient contact interactions. A follow-up study among HCP in El Salvador can also test both anti-spike protein SARS-CoV-2 antibodies and anti-nucleocapsid antibodies to better understand natural infection versus immunity from vaccines.

## Figures and Tables

**Table 1:** Study population characteristics, El Salvador 2021 (*n* = 2176).

Characteristic	*n*	%
Sex		
Male	806	37.1%
Female	1370	62.9%
Age		
Median (range)	43 (18–84)	
Age Group		
≤30	311	14.3%
31–50	1249	57.4%
51–70	608	27.9%
>70	8	0.4%
Residential Department		
Ahuachapán	90	4.1%
Cabañas	74	3.4%
Chalatenango	84	3.9%
Cuscatlán	67	3.1%
La Libertad	199	9.1%
La Paz	73	3.4%
La Unión	61	2.8%
Morazán	47	2.2%
San Miguel	243	11.2%
San Salvador	741	34.0%
San Vicente	54	2.5%
Santa Ana	193	8.9%
Sonsonate	118	5.4%
Usulután	132	6.1%
Geographic Zone		
Central	1292	59.4%
Western	401	18.4%
Eastern	401	22.2%
Number of Household Family Members		
0	44	2.0%
1–3	1061	48.7%
4–6	967	44.4%
≥7	105	4.8%
Primary Job Title		
Medical Staff	487	22.4%
Nursing Staff	491	22.6%
Technical Staff	546	25.1%
Administrative Staff	652	30.0%
Primary Job Function		
Direct Patient Care	1350	62.0%
Administrative	533	24.5%
Auxiliary Services	197	9.1%
Contact with Patient Specimens	96	4.4%
Chronic Disease		
Arterial Hypertension	511	23.5%
Diabetes	184	8.5%
Renal Disease	10	0.5%
Cancer	3	0.1%
Obesity	202	9.3%
Immunological Disease	25	1.2%
Heart Disease	15	0.7%
Chronic Liver Disease	2	0.1%
Autoimmune Disease	9	0.4%
Chronic Respiratory Disease	45	2.1%
Other Endocrine Disease	45	2.1%
Osteopathy/Chondropathy Disease	7	0.3%
Any chronic disease	820	37.7%

**Table 2:** Seropositivity of spike protein SARS-CoV-2 IgG Antibodies, El Salvador 2021 (*n* = 2176).

Characteristic	*n*	Positive IgG Result (n)	Seropositivity (95% Wald CI)	Bivariable OR (95% Wald CI) ^a^
Total	2176	880	40.4% (38.3–42.6)	
Sex				
Male	806	323	40.0% (36.8–43.4)	0.97 (0.82–1.16)
Female	1370	557	40.7% (38.0–43.3)	*Reference*
Age				
Median (range)	43 (18–84)			0.99 (0.98–0.99) *
Age Group				
≤30	311	138	44.4% (39.0–49.8)	1.11 (0.87–1.42)
31–50	1249	522	41.8% (39.0–44.6)	*Reference*
51–70	608	217	35.7% (32.1–39.3)	0.77 (0.64–0.93) *
>70	8	3	37.5% (4.1–70.9)	0.84 (0.20–3.52)
Residential Zone				
Central	1292	545	42.2% (39.4–45.0)	*Reference*
Occidental	401	165	41.1% (37.0–45.3)	0.96 (0.78–1.18)
Oriental	483	170	35.2% (30.6–39.7)	0.74 (0.59–0.94) *
Number of Household Family Members				
0	44	15	34.1% (20.3–47.9)	*Reference*
1–3	1060	396	37.4% (34.3–40.4)	1.15 (0.62–2.14)
4–6	967	414	42.8% (39.7–45.9)	1.45 (0.77–2.72)
≥7	105	55	52.4% (42.9–61.9)	2.13 (1.05–4.33) *
Primary Job Level				
Clinic I	758	300	39.6% (36.1–43.1)	0.96 (0.80–1.16)
Hospital II	1063	431	40.5% (37.7–43.4)	*Reference*
Hospital III	355	149	42.0% (35.5–48.4)	1.06 (0.79–1.42)
Primary Job Function				
Direct Patient Care	1350	555	41.1% (38.6–43.6)	*Reference*
Administrative	533	193	36.2% (31.5–40.9)	0.81 (0.65–1.02)
Auxiliary Services	197	100	50.8% (43.8–57.8)	1.48 (1.10–1.99) *
Contact w/Patient Specimens	96	32	33.3% (25.1–41.6)	0.72 (0.49–1.04)
Number of Jobs				
1	2062	835	40.5% (38.3–42.7)	*Reference*
≥2	114	45	39.5% (29.5–49.4)	0.96 (0.63–1.47)
RT-PCR Positive Result				
No	1377	385	28.0% (25.6–30.3)	*Reference*
Yes	667	441	66.1% (62.0–70.2)	5.03 (4.05–6.24) *
Symptoms in Household Family Members				
No	1413	521	36.9% (34.3–39.5)	*Reference*
Yes	623	302	48.5% (44.5–52.5)	1.61 (1.33–1.96) *
Household Family Members with SARS-CoV-2				
0	1114	362	32.5% (29.5–34.5)	*Reference*
≥1	743	421	56.7% (52.9–60.5)	2.72 (2.21–3.33) *

Abbreviations: CI, Confidence Interval; OR, Odds Ratio; * *p* < 0.05; ^a^ Bivariable logistic regression with a 95% confidence interval adjusting for the complex survey design.

**Table 3:** SARS-CoV-2 IgG seropositivity and self-reported symptoms in the past 3 months, El Salvador 2021 (*n* = 2176).

Symptom	*n*	Positive IgG Result	Seropositivity (95% Wald CI)	Bivariable OR (95% Wald CI) ^a^
Fever	201	123	61.2% (54.6–67.8)	2.54 (1.89–3.41) *
Headache	562	266	47.3% (43.0–51.6)	1.47 (1.21–1.78) *
Sore throat	464	210	45.3% (40.6–49.9)	1.29 (1.05–1.58) *
Body aches	384	200	52.1% (47.2–57.0)	1.78 (1.43–2.21) *
Conjunctivitis	25	5	20.0% (5.4–34.7)	0.37 (0.15–0.92) *
Cough	311	152	48.9% (43.2–54.6)	1.49 (1.16–1.92) *
Shortness of breath	129	74	57.4% (48.9–65.9)	2.07 (1.45–2.96) *
Loss of smell	161	132	82.0% (75.8–88.2)	7.72 (5.06–11.77) *
Loss of taste	138	118	85.5% (79.3–91.8)	9.89 (5.96–16.40) *
Weight loss	70	53	75.7% (66.0–85.4)	4.82 (2.82–8.24) *
Abdominal pain	99	46	46.5% (36.3–56.6)	1.30 (0.85–1.96)
Diarrhea	275	127	46.2% (40.2–52.1)	1.31 (1.01–1.69) *
Skin rash	14	6	42.9% (14.5–71.3)	1.11 (0.35–3.55)
Nasal Congestion	13	9	69.2% (44.0–94.5)	3.34 (1.02–10.96) *
Nausea and vomiting	7	5	71.4% (37.8–100.0)	3.70 (0.72–19.14)
Other	9	5	55.6% (23.0–88.1)	1.85 (0.49–6.92)
None	1250	453	36.2% (33.6–38.9)	0.67 (0.56–0.79) *
Number of Symptoms				
0	1250	453	36.2% (33.6–38.9)	Reference
1	267	111	41.6% (35.5–47.6)	1.25 (0.96–1.64)
≥2	660	316	47.9% (44.0–51.7)	1.62 (1.33–1.96) *

Abbreviations: CI, Confidence Interval; OR, Odds Ratio; * *p* < 0.05; ^a^ Bivariable logistic regression with a 95% confidence interval adjusting for the complex survey design.

**Table 4:** Multivariable risk factor analysis for SAR-CoV-2 spike protein IgG seropositivity, El Salvador 2021 (*n* = 2036).

Variable	Multivariable OR (95% Wald CI)
Age Group (≤30 vs. 31–50)	1.12 (0.86–1.47)
Age Group (51–70 vs. 31–50)	0.81 (0.67–0.99) *
Age Group (>70 vs. 31–50)	1.30 (0.30–5.67)
Residential Zone (Occidental vs Central)	0.97 (0.78–1.20)
Residential Zone (Oriental vs. Central)	0.71 (0.56–0.90) *
Household Family Members (1–3 vs. 0)	1.43 (0.74–2.75)
Household Family Members (4–6 vs. 0)	1.77 (0.91–3.44)
Household Family Members (≥7 vs. 0)	2.56 (1.20–5.46) *
Primary Job Function (administrative vs. patient care)	0.87 (0.69–1.10)
Primary Job Function (auxiliary services vs. patient care)	1.56 (1.12–2.18) *
Primary Job Function (samples vs. patient care)	0.68 (0.47–0.99) *
Number of Symptoms (1 vs. 0)	1.20 (0.90–1.60)
Number of Symptoms (≥2 vs. 0)	1.49 (1.21–1.83) *
Symptoms in Household Family Members (yes vs. no)	1.51 (1.22–1.86) *

Abbreviations: CI, Confidence Interval; OR, Odds Ratio; * *p* < 0.05.

## Data Availability

The data presented in this study are available upon reasonable request from the authors of this manuscript.

## Supplementary Materials

The following supporting information can be downloaded at: https://www.mdpi.com/article/10.3390/idr16030040/s1, Table S1: Sample size for each size of healthcare facility, Table S2: Quotas established for each type of healthcare establishment, Table S3: Distribution of final sample by job function and healthcare facility type (*n* = 2176).
