# Supplementary material for: Seroprevalence of Anti-SARS-CoV-2 IgG Antibodies in Healthcare Personnel in El Salvador Prior to Vaccination Campaigns
Source: Infect Dis Rep. 2024 Jun 7;16(3):531–42. doi: 10.3390/idr16030040 (PMC11203478; doi:10.3390/idr16030040)
Supplement: Supplementary file 1 [file idr-16-00040-s001.zip › idr-2983483-supplementary.pdf]

SUPPLEMENTAL MATERIALS

Supplemental Table S1. Sample size for each size of healthcare facility

Distribution of personnel in each category	Count of personnel in 2013	% Categ.	% Stratum 1	% Stratum 2	% Stratum 3	Hospitals	Establish-ments with more than 30 people	Establish-ments with less than 30 people	Hospitals	More than 30	Less than 30	Total
General Medicine, Specialists, Residents, Social Services Medics	10,110	21.42%	7.50	3.21	1.29	8	4	1	352	152	23	527
Nurses and Nurse Auxillery	11,126	23.58%	8.25	3.54	1.41	8	3	2	352	114	46	512
Technical Staff, Physiotherapists, Anesthetists, Chemistry and Pharmacy, Health Promoters, Environmental Sanitation and Clinical Laboratory, Dentists	11,370	24.09%	8.43	3.61	1.45	8	4	2	352	152	46	550
Administrative staff (Archives, Secretaries, Motorists, Maintenance, Cleaning, Cooking, Laundry, Security, Etc.)	14,584	30.90%	10.82	4.64	1.85	11	4	1	484	152	23	659
TOTAL	47,190		35	15	6	35	15	6	1540	570	138	2,248

Supplemental Table S2: Quotas established for each type of establishment

	MINSAL	ISSS	FOSALUD	ISBM	ISRI	SEM	COSAM	TO TAL
Expected Establishments	819	83	168	57	10	4	2	1,143
Establishments identified from list of staff	593	12	168	57	11	4	1	846
Percentage of establishments	71.65%	7.26%	14.70%	4.99%	0.87%	0.35%	0.17%	100.0 0%
Self-representation								
Self-represented establishments	31	12	0	0	0	0	1	44
Staff identified	14507	7007	0	0	0	0	630	22144
Number of staff to sample (35 samples per establishment)	1085	420	0	0	0	0	35	1540
RANDOM SELECTION								
Establishments with 30 or more staff								
	MINSAL	ISSS	FOSALUD	ISBM	ISRI	SEM	COSAM	TOTAL
Establishments with 30 or more staff	337	34	10	3	9	0	0	393
Staff identified	8510	1474	504	255	0	82	0	10825
Number of establishments to select	30	2	4	1	1	0	0	38
Number of staff to sample (15 samples per establishment)	450	30	60	15	15	0	0	570
Establishments with less than 30 staff								
	MINSAL	ISSS	FOSALUD	ISBM	ISRI	SEM	COSAM	TOTAL
Establishments with less than 30 staff	451	37	158	54	1	4	0	705
Staff identified	4360	0	2344	369	0	0	0	7073
Number of establishments to select	12	1	6	2	1	1	0	23
Sample staff (6 samples per establishment)	72	6	36	12	6	6	0	138
Total Data								
	MINSAL	ISSS	FOSALUD	ISBM	ISRI	SEM	COSAM	TOTAL
Identified establishments	819	83	168	57	10	4	1	1142
Staff identified	27377	8481	2848	624	0	82	630	40042
Number of establishments to select	73	15	10	3	2	1	1	105
Number of staff to sample	1607	456	96	27	21	6	35	2248

Supplemental Table S3. Distribution of final sample by job function and healthcare facility type (N = 2176)

	Administrative	Medical	Nursing	Technical	Total
**Hospital**	484	333	338	341	1496
**Clinic < 30 employees**	31	26	39	48	144
**Clinic ≥ 30 employees**	137	128	114	157	536
**Total**	652	487	491	546	2176
